# A Culinary-Based Intensive Lifestyle Program for Patients with Obesity: The Teaching Kitchen Collaborative Curriculum (TKCC) Pilot Study

**DOI:** 10.3390/nu17111854

**Published:** 2025-05-29

**Authors:** Auden C. McClure, Meredith Fenn, Stephanie R. Lebby, John N. Mecchella, Hannah K. Brilling, Sarah H. Finn, Kimberly A. Dovin, Elsa Chinburg, Jennifer Massa, Kate Janisch, David M. Eisenberg, Richard I. Rothstein

**Affiliations:** 1 Section of Obesity Medicine, Center for Digestive Health, Dartmouth Health, Lebanon, NH 03756, USA; stephanie.r.lebby@hitchcock.org (S.R.L.); hannah.k.brilling@hitchcock.org (H.K.B.); sarah.h.finn@hitchcock.org (S.H.F.); kimberly.a.dovin@hitchcock.org (K.A.D.); 2 Department of Pediatrics, Geisel School of Medicine at Dartmouth, Hanover, NH 03755, USA; 3 Department of Medicine, Geisel School of Medicine at Dartmouth, Hanover, NH 03755, USArichard.i.rothstein@hitchcock.org (R.I.R.); 4 College of Osteopathic Medicine, University of New England, Biddeford, ME 04005, USA; 5 Doctor of Physical Therapy Program, University of Vermont, Burlington, VT 05401, USA; elsa.chinburg@uvm.edu; 6 Department of Nutrition, Harvard TH Chan School of Public Health, Boston, MA 02115, USA; jmassa@hsph.harvard.edu (J.M.); deisenbe@hsph.harvard.edu (D.M.E.)

**Keywords:** teaching kitchen, culinary medicine, preventative health, obesity, lifestyle medicine

## Abstract

**Background**: This study assessed the feasibility, acceptability, and preliminary effectiveness of a teaching kitchen intervention that synergistically provided nutrition education, culinary skills/techniques, mindfulness, physical activity, and behavior change strategies. **Methods**: Non-randomized pilot study of 16 weekly 2 h hands-on virtual culinary sessions. Curbside grocery pickup assured food access/consistency. Qualitative interviews and pre/post-anthropometrics (BMI, waist circumference), labs (fasting glucose, insulin, lipids, HbA1c, ALT), and health habits surveys assessed program impact. **Results**: The program was successfully implemented from January to May of 2022. Of 56 participants screened, 13 (23%) enrolled, and 12 (92%) completed the program (mean age 51 years; 92% female; 92% white) with an average of 15.4 of 16 (96%) sessions attended and 100% completing assessments. Satisfaction with the program and with virtual cooking was high (100% and 92% satisfied-very satisfied). Days/week main meal was prepared from scratch increased from 3.8 to 5.9 (*p* < 0.05). Sense of well-being and three core mindfulness items (satiety, snacking, and food appreciation) improved (*p* ≤ 0.05). Confidence in 13 culinary skills/techniques improved (*p* < 0.05), as did diet recall and daily exercise, with variable significance. Labs improved LDL significantly (*p* < 0.05); anthropometrics did not. **Conclusions**: This teaching kitchen program was feasible, very well accepted, and suggested potential efficacy in improving health habits and metrics. Larger studies with randomization are needed.

## 1. Introduction

Diet is a modifiable risk factor and determinant of chronic disease [1,2,3]. Dietary patterns rich in fruits and vegetables, whole grains, unsaturated fats, low-fat dairy, nuts, beans, and legumes have been shown to decrease the risk of chronic diseases and promote long-term health, as has lower intake of trans fats, processed and red meats, excess sodium, refined grains, and sugar-sweetened beverages [4,5,6]. These findings are in line with recommended dietary guidelines, and yet national adherence to guidelines remains low [7,8]. While lifestyle change, including better diet quality, can improve health outcomes and prevent chronic disease, health professionals and healthcare systems have struggled to find modalities to assure equitable access to healthy foods and support patients in sustainable change [9,10]. Nutrition insecurity leads not only to poor health outcomes but also to lost productivity and increased healthcare spending [11,12,13]. Given the rising prevalence of obesity, type 2 diabetes, and other metabolic diseases such as metabolic dysfunction-associated steatohepatitis, it is essential to identify novel lifestyle interventions and strategies that can be integrated into primary or specialty care or disseminated widely in community settings to promote recommended health behaviors to impact chronic disease and address nutrition inequities [9,11,14,15,16,17,18,19,20].

Food is Medicine (FIM) programs have emerged in response to this need. There is growing evidence to support the use of FIM interventions, including medical nutrition therapy (MNT) [21], Medically Tailored Meals (MTM) [22,23,24], and Produce Prescription Programs (PPP) [25,26,27,28,29,30,31] to promote food access and assure nutrition security as key social determinants of health. These programs, if implemented widely, have the potential for significant healthcare cost savings and improved health outcomes, making food and nutrition security a national priority [15,18,19,32,33,34,35,36,37]. There is also growing recognition that FIM initiatives aimed at addressing food access must be paired with nutrition and culinary education to provide both the knowledge and skills to independently plan, shop for, and prepare nutritious food and meals [18,19,38]. Beyond traditional lifestyle counseling, evidence supports teaching kitchen interventions as a means to translate evidence-based nutrition and lifestyle recommendations into daily lifestyle habits, allowing for greater sustainability of health-promoting lifestyle patterns through practice and adaptation, thus potentially impacting cardiometabolic outcomes [39,40,41,42,43,44,45,46,47,48,49,50,51]. Through experiential learning, teaching kitchens can provide knowledge, skills, and self-efficacy to support participants in adopting daily lifestyle habits that fit with their social and cultural context. However, there is a need for rigorous testing of best practices to support their use and assess relevant outcomes [52,53,54].

To our knowledge, no studies have tested the impact of a referral-based virtual teaching kitchen intervention that synergistically provides basic cooking skills and techniques with an evidence-based curriculum that includes nutrition guidance, mindfulness, exercise, and behavior change strategies to patients as an adjunct to chronic disease management in the primary or tertiary care setting. We proposed in this pilot study to test, in a population of adults with obesity and no diabetes, the feasibility, acceptability, and preliminary effectiveness of the Teaching Kitchen program, with 16 modular classes taught virtually by a chef, dietitian, and health coach. Through future adaptation to clinic and community settings, Teaching Kitchen programs have the potential to impact population health by disseminating evidence-based lifestyle interventions nationally, adding to evidence supporting FIM interventions as part of regional healthcare systems.

## 2. Materials and Methods

### 2.1. Study Design and Recruitment (Figure 1)

The study was a prospective single-arm pilot feasibility study that took place at Dartmouth Health in Lebanon, NH, from January to May of 2022. A cohort of adults with obesity was recruited at a rural academic medical center with the goal of capturing those at increased metabolic risk. Patients with severe illness, type 2 diabetes, or currently taking diabetes or anti-obesity medications were excluded. A clinic-based recruitment strategy (referral by health care providers) was used initially, with a shift to a broader institutional email and social media-based recruitment to increase the number of eligible participants reached. Medical clearance for participation was obtained from participants’ primary care providers. A research assistant completed an initial screening phone call prior to a virtual video information and consent visit. Due to COVID-19 restrictions, the only in-person visits were for assessment visits (anthropometrics and labs) and for exit interviews. Surveys and consent were administered virtually via REDCap [55,56]. Exit interviews were recorded using two separate devices and transcribed using Dedoose [57], with any identifiers removed manually. Incentives for participants in the study included the hands-on teaching kitchen experience with recipe ingredients, a cast iron pan, and gift certificates provided for completion of assessment visits.

**Figure 1:**
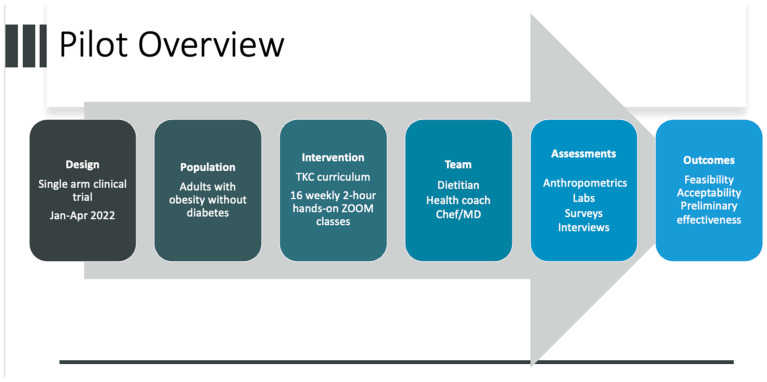
Pilot Overview.

### 2.2. Development of the Teaching Kitchen Collaborative Curriculum (TKCC)

**Figure 2:**
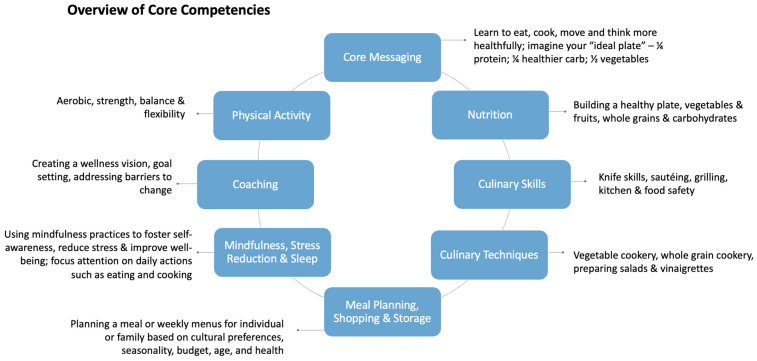
Overview of teaching kitchen core curricular domains.

The Teaching Kitchen Collaborative (TKC) [58,59] is a global network of medical professionals, chefs, dietitians, and food system experts working to improve the health and well-being of a variety of populations through experiential teaching kitchen education. In 2016, the TKC brought together thought leaders to develop core competencies for teaching kitchens. Competencies were developed iteratively through a series of collaborative meetings with subject matter experts in the teaching kitchen and self-care domains. Whiteboard brainstorming with thematic analysis led to an initial draft of competencies, followed by serial revisions and then final review by experts. A set of core competencies was successfully developed across multiple domains (nutrition knowledge, culinary competency, exercise, mindfulness, and strategies for behavior change) with the goal of informing TKC educational and research programs directed at a range of populations and disease states (Figure 2) [14,40,59]. These core principles guided the development of the specific curriculum implemented in this pilot study, which was developed by the Dartmouth Health Culinary Medicine Program in collaboration with Harvard researchers and tailored toward patients with obesity looking to improve health habits and prevent or address metabolic disease.

### 2.3. Program Description/Study Intervention

Study participants attended 16 weekly, 2 h hands-on virtual teaching kitchen classes covering evidence-based dietary recommendations, the link between lifestyle and health, cooking skills and techniques, shopping and meal planning, mindfulness and stress reduction skills, sleep optimization, activity coaching, and strategies for behavior change (Figure 2). For the pilot, the sessions covered core education domains with classes organized around nutrition topics with paired recipes, culinary skills, and techniques. Mindfulness, movement, strategies for lifestyle change, and goal setting were interwoven into the curriculum. Dietary counseling focused on healthy, balanced, “plant-forward” eating patterns, intentionally shifting away from “diets” or calorie restriction, and guided by the Healthy Eating Plate from the Harvard T.H. Chan School of Public Health’s Nutrition Source [60]. Curriculum content also addressed food access, preparation, and storage to enhance nutrition security. During class, the chef first demonstrated class recipes, highlighting new skills and techniques prior to participant cooking. Focus then shifted to participants’ kitchen workstations, which allowed for close virtual monitoring of participant cooking by the teaching kitchen team. Nutrition topics were presented by the dietitian in a 20 min PowerPoint (summarized in a key Take Home Points handout), and recipe-specific nutrition, meal planning, and shopping points were highlighted throughout the class with the chef and dietitian teaching in tandem. The health coach led mindfulness and movement activities and provided coaching throughout the class to set goals, address barriers, and celebrate successes through shared group experiences. Class ended with sharing of final plates, a mindful moment, and tasting, emphasizing the exploration of new flavors and the shared experience of cooking and eating together. See Appendix A for a more detailed description of curriculum domains and Appendix B for an outline of the weekly curriculum (class topic, skills, and recipes).

### 2.4. Teaching Kitchen Facilities

Due to COVID-19 restrictions, classes were taught virtually (via video platform) by a chef, dietitian, and health coach with participants cooking in their home kitchens. A research assistant was available to help with technical issues, class monitoring, and documentation. The chef taught from a home teaching kitchen with both a computer and an iPhone connected to the video platform. A large monitor allowed for observation of participants, and the iPhone was positioned on a tripod to allow for demonstration of the workspace and cooktop. Classes were scheduled as group video visits through the EPIC electronic medical record (EMR), allowing for the use of standard appointment reminders and EMR communication. Staples boxes, with shelf-stable ingredients, were assembled by a research assistant and provided to participants monthly, and fresh groceries were offered weekly via curbside pickup at a local cooperative grocery store. A checklist of basic cooking equipment was reviewed with participants prior to the study start, and missing items were offered. Each participant was provided with a cast iron pan as part of study incentives, and many recipes were tailored to its use.

### 2.5. Assessment and Measures

Demographics, including age, gender, race, household income, highest level of education, rural vs. urban area, and distance to the closest grocery store, were collected as part of screening eligibility and on baseline surveys. The feasibility of conducting this intensive lifestyle program was assessed through recruitment, retention, and class attendance rates. Acceptability was assessed with participant feedback surveys and semi-structured exit interviews. Qualitative interviews were conducted virtually at the end of the intervention to gather more information about the participants’ experiences and to receive feedback on the intervention and study design. The semi-structured interview guide included open-ended questions asking about motivation to join the study, overall impressions, feedback on the structure and content of the program and video platform, facilitators and barriers to incorporating skills learned into daily life, and open-ended final thoughts. To determine the preliminary effectiveness of the intervention, a study visit was scheduled at baseline and after the final class, at which a research assistant obtained anthropometrics (height, weight, body mass index (BMI), and waist circumference). Participants were then directed to the hospital laboratory for a study blood draw to obtain fasting metabolic labs (hemoglobin A1C (HbA1c), alanine aminotransferase (ALT), fasting glucose, insulin, and lipid panel). Finally, self-reported health behaviors were assessed using a novel Teaching Kitchen Survey (TK Survey) and additional validated survey instruments (described below). Medications were reviewed at baseline, for eligibility, and at the end of classes to avoid confounding due to the initiation of obesity, diabetes, or cholesterol medications. Finally, we measured fidelity of program implementation (percent of planned classes and assessment visits offered as planned), as well as barriers and facilitators to effective implementation of the teaching kitchen curriculum in a community-based setting.

The TK Survey was developed iteratively by the Teaching Kitchen Collaborative research team as a singular assessment tool that could be used to assess a range of teaching kitchen programs across various settings and populations. Items were included to assess each of the core curricular domains:

- Health and well-being were assessed through two survey items rating perceived overall health [61,62] and well-being [63].
- Meal planning and food shopping were captured through 5 items asking about meals prepared at home and use of nutrition labels [64].
- Food insecurity was assessed using the Hunger Vital Signs [65,66].
- Culinary skills and techniques were assessed using a modified version of the Cooking with a Chef survey [67]. Fifteen items from this survey measuring confidence in culinary skills were combined with additional items measuring confidence in using/adapting recipes and planning meals [68].
- Dietary patterns were captured using 4 items from the Prime Diet Quality Score 30 (PDQS-30) [69] for intake of whole grains, refined grains/baked goods, salty snacks, and sugary drinks (PDQS-30 questions 20, 21, 22, 23). Two items combined several questions from the PDQS-30 to measure overall fruit and vegetable intake:
  ○ Fruit: Over the past month, how often did you eat fruits (include only whole fruit, not juices)?
  ○ Vegetable: Over the past month, how often did you eat vegetables (including fresh, frozen, or canned, eaten separately or as part of a mixed dish)?
- Mindful eating was assessed using 3 items from the Mindful Eating Questionnaire [70]. These focused on satiety awareness, mindless snacking, and sensory food appreciation, topics highlighted in the curriculum.
- Movement was assessed with the Exercise Vital Sign [71].
- Sleep hour and quality were captured with two survey items querying the amount and quality of sleep [72,73].

Additional, longer validated survey instruments were also implemented as part of the pilot study to test the validity of the TK Study survey instrument in anticipation of a larger randomized controlled trial, allowing for more rigorous evaluation with greater power. In addition to the 5 core items included in the TK Survey, a modified version of the full Prime Diet Quality Score (PDQS-30) dietary assessment [69] was used to assess self-reported dietary patterns. The PDQS-30 was minimally adapted to better reflect commonly consumed foods in this study population. Five items were added assessing consumption of whole milk, cheese, fermented foods, salty snacks, and alcohol (Appendix C). In addition, minor modifications to language were made to improve contextual relevance (i.e., “mixed” dishes instead of “composite” dishes, potatoes instead of “white roots and tubers”). Modifications were reviewed and approved by the original developers of the instrument, who confirmed that the proposed substitutions and additions were appropriate.

Similarly, in addition to the three core items in the TK survey, the full Mindful Eating Questionnaire (MEQ) [70] was deployed to capture mindful eating behaviors across several sub-categories, including feelings of disinhibition, awareness, external cues, emotional response, and distraction related to food and eating patterns with a total summary score. This was modified to allow respondents to choose “not applicable” for each instead of selected items, with only scored responses included in score denominators per scoring algorithm. The Medical Outcomes Study (MOS) Short Form 20 (SF20) [61,62] was used to assess health-related quality of life (HRQOL). Finally, a modified version of the PROMIS APA DSM5 Sleep Disturbance Scale [74] was used to assess sleep patterns.

### 2.6. Statistical Analysis

Descriptive statistics (means, standard deviation) were computed at baseline. Paired *t*-tests were used to assess for change in anthropometrics, lab values, and survey measures over the sixteen-week program. As this was a small pilot study (*n* = 12), we used paired *t*-tests assuming approximate normality to enhance power and sensitivity. For core PDQS-30 survey items, graphic displays were used to illustrate the shift in pre/post frequency of food consumption, with significant change via *t*-test also noted. For participant interviews, thematic inductive coding methods were used to analyze qualitative feedback and identify emergent themes. Two researchers conducted open and then focused coding and then independently reviewed the de-identified interview transcripts and manually conducted an initial round of thematic coding. Transcripts were studied line by line, and relevant codes were assigned to specific words or phrases. A codebook was created based on the reliability of codes, and codebook definitions were reconciled between coders. Codes were then grouped and refined based on mutual consensus between the two reviewers into major themes.

## 3. Results

### 3.1. Sample Description

The mean age of the 12 participants completing the study was 51.6 years; 92% were female, and 92% reported white race. In this sample, 42% reported yearly household income ≥ 100,000, 50% had attained a bachelor’s or graduate degree, 83% reported living in a rural area, and 83% reported access to a grocery store within 10 miles.

### 3.2. Feasibility

Initial efforts at enrollment through clinic-based referral were challenging due to the limited number of eligible and interested patients presenting to clinics as well as the time burden on clinic teams with screening. Recruitment improved once broader email and social media recruitment strategies were implemented, allowing participants to self-refer, with 13 of 56 screened subjects enrolling (23%). Over the course of the intervention, 12 of 13 (92%) participants completed the 16-week program, with program completers attending an average of 15.4 (96%) sessions and successfully completing class recipes. Implementation of the intervention was successful, with 100% of virtual classes and assessments offered as planned.

### 3.3. Acceptability

**Figure 3:**
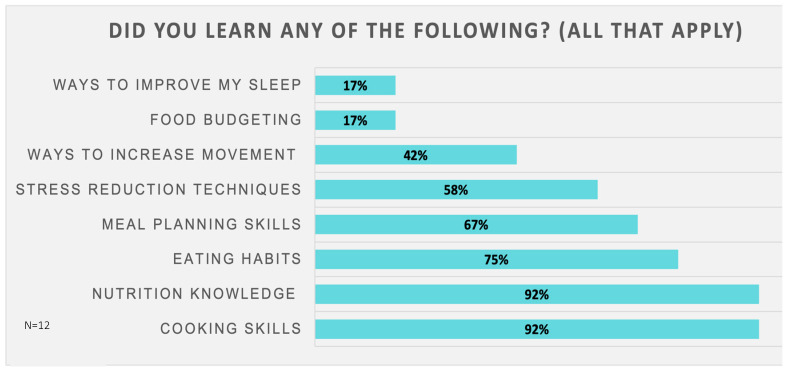
Feedback Survey: Self-report of areas of lifestyle learning.

**Figure 4:**
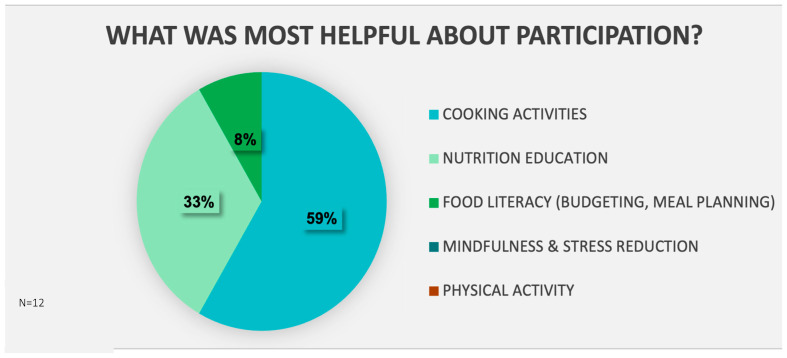
Feedback Survey: Self-report of most helpful areas of participation.

Participant feedback was elicited through a final survey and from semi-structured interviews. On feedback surveys, there was high satisfaction (Likert 1–5: very dissatisfied–very satisfied) with the program overall as well as with the virtual cooking experience (100% and 92% satisfied/very satisfied, respectively). The majority of participants (92%) felt that the difficulty of recipes chosen for the class was “just right” (Likert 1–5: too easy–too difficult), 100% reported that they were successful/very successful with cooking the recipes during class (Likert 1–5: Not successful–very successful), and 92% reported that they would continue to make the class recipes (or variations) in the future (Yes, No, Not sure). The majority of participants reported having access to food used in the recipe and equipment used in the class. In Figure 3, when asked to choose ALL that apply, participants reported learning about multiple topics, with cooking skills and nutrition knowledge being most commonly reported (92%), followed by eating habits (75%), meal planning skills (67%), stress reduction techniques (58%), and ways to improve movement (42%). Fewer participants reported learning about food budgeting and ways to improve sleep (17% each). In Figure 4, when asked to choose what was MOST helpful, participants found cooking activities to be most helpful (59%), followed by nutrition education (33%), and then food literacy (8%). Mindfulness/stress reduction and physical activity components of the program were not chosen (Figure 4), which reflects the lesser emphasis of these in the curriculum.

In qualitative analysis of semi-structured interviews, a total of 26 themes emerged, with the five most prominent (those discussed the most) being goals for participation in the program, behavior change/impact on health that resulted from participation, challenges to participation in the program, feedback about program logistics, and feedback about program content (curricular domains). Many participants reported seeking to improve their current or future health through better cooking skills and techniques, a better understanding of what to eat (nutrition knowledge), as well as through more health-based cooking and recipes. As a result of participating, many reported being able to make small sustainable behavior changes over the course of the study, such as cooking more at home, integrating recipes and techniques learned into their daily lives, and incorporating more vegetables and other healthy ingredients into their meals and those of their family. Regarding program logistics, many participants appreciated that the study was conducted remotely, as this allowed them to cook in their own kitchen, where they were comfortable, and could fit the classes into busy schedules. Participants appreciated having all ingredients provided, and yet some participants found grocery pickup logistically challenging. Additional feedback about challenges to participation related primarily to virtual participation and included unstable Wi-Fi connections and difficulty with finding the correct camera setup to both watch the chef demonstrations and to show home work stations while cooking. Remote discussion was more challenging, with participants having to unmute in the midst of cooking, take turns talking, or use the chat feature. Lack of personal connection with others in the group was also expressed as a downside of virtual classes, including initial discomfort with sharing personal goals and challenges. When asked, some participants felt that this program might benefit from a hybrid approach with monthly classes offered in person at the teaching kitchen. Regardless, hearing from other participants about goals, challenges, and successes was helpful and both validating and inspiring. Participants felt that while the length of the program (16 weeks) was initially daunting due to the time commitment, and that sometimes it was a lot to take on, they ultimately found it to be beneficial, enjoyed the consistency of meeting on a weekly basis, and felt it went by quickly. Some felt sessions could be shorter to decrease conflict with other evening commitments. Feedback about the course was overall very positive, with participants reporting that they enjoyed the content and instructors and looked forward to class every week, that they were empowered by what they learned and had new tools, even if still working to make changes stick. Many felt more confident in the kitchen and able to incorporate recipes, combinations, and flavors into family cooking routines. Participants reported learning not only about cooking but also about nutrition, mindfulness, activity, and how to make this happen in day-to-day life. Some noted the exercise component could be more robust. A few felt they would lose more weight, but their focus shifted to health goals. Many shared examples of success with trying new foods, movement routines, and mindfulness. Representative quotes for the five themes are presented in Table 1.

### 3.4. Effectiveness

#### 3.4.1. Teaching Kitchen Survey (Table 2)

**Figure 5:**
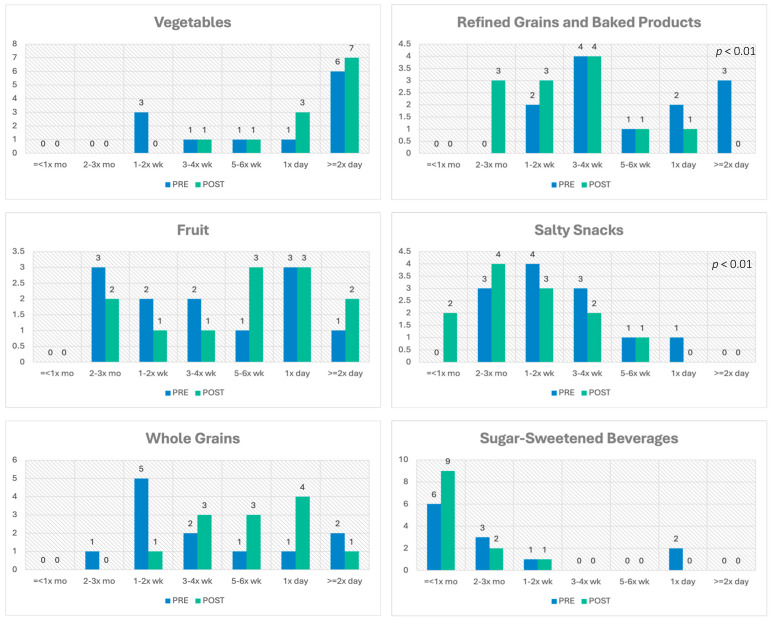
Bar graphs show change in reported frequency of consumption of 6 core dietary items using questions adapted from the Prime Diet Quality Score (PDQS-30) survey [69]. *p*-Values denote significant change, using paired *t*-tests, from baseline to post-class assessment.

A core objective of the study was to guide participants in preparing meals at home from whole ingredients. Over the course of the 16-week program, the reported frequency of main meals/dinners prepared at home from scratch increased significantly from 3.8 to 5.9 days per week. Confidence in preparing the main meal from scratch and use of nutrition facts when shopping both increased but did not reach significance. Food insecurity was low in this small sample (92% reported never true), with no significant change seen over the course of the program. Improvement was seen in culinary skills and techniques over the course of the program, with significant improvement in self-reported confidence in 13 of 19 items queried (steaming, sautéing, stir-frying, grilling, poaching, baking, roasting, simmering, preparing root vegetables, planning nutritious meals, preparing a meal from items on hand, and adapting a recipe to use fresh whole ingredients that are on hand). Non-significant improvements were seen in confidence in knife skills, using basic cooking techniques, preparing fruit and fresh/frozen green vegetables, following a recipe (skills that had higher reported confidence scores at baseline), and using herbs and spices. Dietary recall items capturing consumption of food groups emphasized in the curriculum were included in the TK Survey. At the end of the teaching kitchen program, self-reported frequency of consumption of refined grains/baked goods and salty snacks decreased significantly, and there was a non-significant decrease in sugary drinks and an increase in fruits, vegetables, and whole grains consumed (Figure 5). Four mindful eating items were assessed as part of the TK Survey: Frequency of eating mindfully and with intent, stopping eating when full, snacking without noticing, and taking a moment to appreciate before eating all showed improvement at the end of the program with variable significance. Both self-reported days per week and minutes per day of exercise increased (2.67 to 3.58 days per week, 21.7 to 26.7 min per day) but did not reach significance. No change was seen in the self-reported amount or quality of sleep over the course of the study. No significant change was seen in self-rated overall health and work-life balance, but sense of well-being improved significantly (*p* < 0.05).

**Table 2:** Self-reported health behaviors: Baseline and post-intervention (*n* = 12).

Survey Question	Baseline Mean (SD)	Post Class Mean (SD)	*p* Value
**Health and wellbeing**			
In general, would you say your health is? [Likert 0–5, poor, fair, good, very good, excellent] [62]	3.0 (0.74)	2.9 (0.67)	0.59
How would you rate your current “work-life” balance? With 1 being poor and 10 being very good [50]?	5.8 (2.66)	6.0 (2.66)	0.83
How would you rate your current sense of well-being? With 1 being poor and 10 being very good [63]	6.1 (2.12)	7.7 (2.27)	0.02
**Meal planning and food shopping**			
The last time you went grocery shopping, did you use the nutrition facts label to guide your choices? [Likert 0–3, never/rarely, sometimes, often, usually/always] [75]	1.0 (1.13)	1.75 (1.14)	0.02
How many days a week do you prepare your main meal/dinner from “scratch” using fresh ingredients (including fresh, frozen, or canned produce)-ok to count your own leftovers?	3.83 (2.33)	5.92 (1.68)	<0.01
How confident do you feel about being able to prepare your main meal/dinner from “scratch” using fresh whole ingredients (including fresh, frozen, or canned produce)? [Likert 0–4, not at all confident, not very confident, neutral, confident, extremely confident]	3.33 (0.89)	3.67 (0.49)	0.10
The next few questions will ask you about cooking at home. By cooking at home, I mean a meal prepared at home from scratch using vegetables, meats, grains, or other fixings [50].			
Thinking about the past 7 days, on how many days did you, personally, cook lunch at your home?	2.83 (1.95)	3.92 (2.78)	0.22
Thinking about the past 7 days, on how many days did you, personally, cook dinner at your home?	3.08 (1.62)	5.33 (1.44)	<0.01
**Culinary skills and techniques—adapted from the Culinary Attitude and Self-Efficacy Scale, Cooking Assessment Questionaire.** [67,68]			
Indicate the extent to which you feel confident about performing each of the following activities [Likert 0–4, not at all confident, not very confident, neutral, confident, extremely confident]			
Using knife skills in the kitchen	3.33 (0.65)	3.67 (0.49)	0.10
Using basic cooking techniques	3.33 (0.65)	3.58 (0.67)	0.19
Steaming	3.00 (0.60)	3.67 (0.65)	0.03
Sautéing	2.92 (1.24)	3.75 (0.45)	0.03
Stir-frying	2.83 (1.03)	3.83 (0.39)	<0.01
Grilling	2.42 (1.24)	3.25 (0.97)	0.02
Poaching	1.25 (0.87)	2.5 (1.09)	<0.01
Baking	3.08 (0.90)	3.5 (0.67)	0.02
Roasting	2.92 (1.0)	3.67 (0.65)	0.02
Stewing	2.08 (1.56)	3.25 (1.06)	0.01
Simmering	2.83 (1.12)	3.67 (0.65)	0.03
Preparing fresh or frozen green vegetables (e.g., broccoli, spinach)	3.33 (1.16)	3.75 (0.62)	0.30
Preparing root vegetables (e.g., potatoes, beets, sweet potatoes)	3.00 (1.04)	3.75 (0.45)	0.02
Preparing fruit (e.g., peaches, watermelon)	3.58 (0.67)	3.83 (0.39)	0.28
Using herbs and spices (e.g., basil, thyme, cayenne pepper)	2.75 (1.29)	3.5 (0.67)	0.08
Planning nutritious meals [in advance]	2.17 (1.34)	3.08 (0.90)	0.03
Following a recipe (ex., salsa from tomatoes, onion, garlic, peppers)	3.25 (0.62)	3.67 (0.49)	0.05
Preparing a meal from items on hand (ex., in pantry and refrigerator)	2.75 (1.14)	3.42 (0.67)	0.04
Adapting a recipe to use fresh whole ingredients that are on hand	2.42 (1.24)	3.25 (0.75)	0.03
**Mindful eating**			
How often do you eat mindfully, with thoughtfulness and intention? [Likert 0–3, never/rarely, sometimes, often, usually/always]	1.00 (0.74)	1.42 (1.08)	0.14
Mindful Eating Questionaire—3 items: [70]			
I snack without noticing that I am eating. [Likert 1–4, never/rarely, sometimes, often, usually/always]	2.58 (0.79)	1.83 (0.72)	0.02
I stop eating when I am full even when eating something that I love. [Likert 1–4, never/rarely, sometimes, often, usually/always]	2.0 (0.95)	2.58 (1.08)	0.05
Before I eat, I take a moment to appreciate the colors and smells of my food. [Likert 1–4, never/rarely, sometimes, often, usually/always]	1.5 (0.80)	2.5 (1.0)	<0.01
**Food security and access**			
How far is the closest grocery store from where you live?	83% <10 miles	83% <10 miles	
Hunger Vital Signs: [65,66]			
Within the past 12 months we worried whether our food would run out before we got money to buy more. [Likert 0–4, often true, sometimes true, never true, do not know/not sure]	92% never true	100% never true	0.34
Within the past 12 months, the food we bought just did not last, and we did not have money to get more. [Likert 0–4, often true, sometimes true, never true, do not know/not sure]	100% never true	92% never true	0.34
**Movement—Exercise Vital Signs** [71]			
On average, how many days per week do you engage in moderate to vigorous physical activity? (like a brisk walk)?	2.67 (2.2)	3.58 (2.4)	0.10
On average, how many minutes per day do you engage in physical activity at this level?	21.7 (17.5)	26.7 (17.8)	0.24
**Sleep**			
How many hours do you usually sleep in a 24 h period (including naps)? [Likert 1–5: Less than 5 h, 5–6 h, 6–7 h, 7–8 h, 8+ h] [73]	3.58 (0.79)	3.33 (0.65)	0.19
During the past month, how would you rate how your sleep quality overall? [Likert 1–4: very good, fairly good, fairly bad, very bad] [72]	2.08 (0.67)	1.92 (0.52)	0.34

Note: boldface indicates significance.

#### 3.4.2. Additional Survey Instruments (See Appendix C)

Additional surveys further assessed key domains addressed in the curriculum, including mindful eating and dietary choices. Sub-score results for the full Mindful Eating Questionnaire (MEQ) improved at the end of the study, with awareness, disinhibition, and emotional scores reaching significance. In addition, the total MEQ score increased significantly from 11.94 to 13.68 (*p* < 0.05). On diet recall, assessed through modified PDQS-30, increased mean frequency of consumption of fruit (citrus, deep orange, other), non-starchy vegetables (leafy greens, cruciferous, other), beans and legumes, nuts and seeds, eggs, poultry, fish, low- and full-fat milk, fermented foods, and liquid oils was reported, but the change was not statistically significant. Only the increase in the consumption of cruciferous vegetables, fish, and oils reached significance. Conversely, decreased consumption of red and processed meats, cheese, sweets and ice cream, and fried foods was seen, with processed meat decreasing significantly (results for whole grains, refined grains, salty snacks, and sweet beverages are reported as part of the TK Survey). No significant change was seen in the Medical Outcomes Study (MOS) Short Form 20 (SF20) [61,62], which was used to assess health-related quality of life (HRQOL), or the modified PROMIS APA DSM5 Sleep Disturbance Scale [74] (data not reported).

#### 3.4.3. Anthropometrics and Biometrics (Table 3)

Minimal non-significant decreases in weight, BMI, and waist circumference were seen over the course of the program. Lab values all improved, with only the decrease in mean LDL level reaching significance.

**Table 3:** Anthropometric and biometric measures: Baseline and post-intervention (*n* = 12).

Measure	Baseline Mean (SD)	Post Class Mean (SD)	*p*-Value
BMI (kg/m^2^)	35.6 (2.5)	35.4 (2.6)	0.53
Weight (kg)	96.1 (10.2)	95.6 (9.6)	0.50
Waist Circumference (cm)	111.8 (8.2)	111.4 (8.0)	0.57
Fasting Glucose	102.5 (12.7)	101.3 (12.4)	0.64
HbA1c (mmol/mol)	5.4 (0.4)	5.3 (0.4)	0.20
Total Cholesterol (mg/dL)	200.2 (46.2)	194.6 (42.5)	0.16
Triglycerides (mg/dL)	159.3 (90.3)	157.7 (106.3)	0.90
LDL (mg/dL)	120.5 (31.9)	113.3 (26.2)	**0.03**
HDL	47.8 (6.6)	49.9 (7.8)	0.13
ALT	25.3 (16.7)	24.5 (13.7)	0.71
Insulin	17.8 (14.0)	16.5 (7.0)	0.66

Note: boldface indicates significance. Abbreviations: BMI, body mass index; HbA1c, hemoglobin A1C; LDL, low-density lipoprotein; HDL, high-density lipoprotein; ALT, alanine aminotransferase.

## 4. Discussion

In this pilot study, we demonstrated, in a small convenience sample of adults with obesity, that a virtual intensive teaching kitchen lifestyle intervention with curbside grocery pickup and hands-on culinary instruction at home was not only feasible but also very well accepted and potentially efficacious in improving health habits and biometric outcomes. Over the course of this hands-on 16-week program, we saw increased confidence in 19 culinary skills and techniques, with 79% reaching significance. Importantly, the intervention led to a significant increase in main meals or dinner prepared at home from scratch (*p* < 0.01), which has been linked in the literature to better dietary intake and health [76]. This finding is consistent with participant reports of greater adherence to dietary recommendations, with significant decreases in intake of refined grains and sugar-sweetened beverages. In addition, increased consumption of fruits, vegetables, and whole grains and decreased intake of salty snacks were reported, but the change was not statistically significant. Participants reported increased confidence in planning, shopping for, and preparing recipes at home, using nutrition labels, and adapting recipes to preferred or available ingredients—all skills that were emphasized in the curriculum.

As seen in other teaching kitchen programs, participants reported, with variable significance among related measures, an increase in mindful eating [16,41]. Despite a more limited focus in the curriculum on daily movement, we were able to demonstrate a non-significant increase in days per week and minutes per day of exercise. Both anthropometric and metabolic outcomes improved but, excepting LDL, were not statistically significant in this small sample. These findings add to a growing body of literature supporting the use of teaching kitchens to provide the knowledge and skills necessary to change daily habits that can impact health outcomes. Finally, in exit interviews, participants report not only increased confidence but also more enjoyment in cooking and eating healthy foods.

Moore et al. and Eisenberg et al. assert that the combination of didactic plus experiential learning can be especially impactful in changing behavior and that the teaching kitchen, with a team of culinary educators, has the capacity to move beyond traditional Medical Nutrition Therapy (MNT) or cooking classes to support behavior change [16,43,50]. An important principle of the curriculum is the “technique-driven, recipe-inspired” approach, with a focus on both skills and techniques, like making a soup, stew, salad meal, or taco, but encouraging participants to adapt recipes to personal taste, culturally familiar ingredients, what is on sale, in season, or easily accessible. The additional curricular focus on shopping, meal planning, and repurposing of ingredients provides essential skills in addressing barriers related to cost, time, access, and cultural relevance that may impact nutrition security. Learning to make healthy and delicious meals may help with the sustainability of behavior change, allowing for gradual shifts in eating patterns, as opposed to dieting, with a focus on enjoying food and the preparation and sharing of it [40,77].

### 4.1. Limitations

Because the study was designed as a pilot feasibility study in anticipation of a larger randomized trial, we acknowledge a number of limitations, including a small, homogeneous sample and the lack of a control group, which limits our ability to fully attribute observed changes to the intervention alone. In addition, lack of longitudinal follow-up after the intervention period precludes assessment of longitudinal changes in anthropometrics, biometrics, and health behaviors and should be considered in designing future study protocols. Reports of food insecurity and other socioeconomic barriers were low in this group of participants, as was racial diversity; future studies should explore how to adapt teaching kitchen programs to assure accessibility and cultural relevance in more diverse populations. Beyond biometrics, all measures were self-reported and hence subject to bias. Objective assessments of culinary skills through observation or photo-documentation of recipe completion and more rigorous assessment of dietary intake and physical activity using tracking devices would be important to consider in future studies. Finally, we examined pre/post outcomes but did not control for potential confounding variables in the analysis. However, we did attempt to limit confounding by excluding those with extreme obesity, serious illness or diabetes, and those who had undergone bariatric surgery or were taking diabetes or anti-obesity medications beyond Metformin. We monitored for new medications, including cholesterol-lowering agents, as well as participation in other high-intensity lifestyle programs; however, more rigorous tracking of confounders would be a next step. At the time of the study, anti-obesity medications were less widely used. These would be important to control for in future studies acknowledging the widespread use of medication and bariatric surgery at this time.

### 4.2. Future Directions

Further research should expand upon this study, not only by testing the curriculum in a larger and more diverse sample [14] but also by adapting the current curriculum to other populations, such as children or those with diabetes. Greater intensity of lifestyle interventions has been linked to improved outcomes, yet programs are time-intensive and require significant participant and staff commitment with variable response [78]. Given the intensity of resources needed to implement a longitudinal lifestyle program, efforts to explore minimal effective doses (varying length or intensity of intervention), to offer programs virtually (less staff needed, lower transportation barriers), and to link teaching kitchens to food access programs (to provide ingredients and help participants to use them) are all areas of research needed to limit both the cost of offering programs and barriers to participation. The virtual delivery of this teaching kitchen curriculum was well accepted during COVID-19, but future studies exploring the differential impact of in-person or hybrid curricula are needed to maximize the impact of FIM programs. Finally, in an era of rapidly evolving anti-obesity medications, the impact of a teaching kitchen program in combination with newer anti-obesity medications could be assessed, as with other multi-pronged approaches to treatment, like diabetes and hypertension. Defining the role of FIM in this new era will be critical to ensure optimal future health outcomes.

## 5. Conclusions

This teaching kitchen-based intensive lifestyle program was feasible, very well accepted, and suggested potential efficacy in improving health habits and metrics. Teaching kitchens may be an effective tool for engaging participants in lifestyle change to prevent or address chronic disease, as a single modality or in conjunction with other medical therapies; however, larger studies in additional populations, with randomization and greater power to detect change, are needed to refine curricula and further test this innovative model.

## Figures and Tables

**Table 1:** Semi-structured interviews: Representative quotes for qualitative themes.

Theme	Selected Quote
Goals	
	I am not a good cook and … I saw it as a good opportunity to learn, which I learned a lot.
	I have … kids and I need to be around for a while.
	I just wanted to learn how to get more vegetables in my diet [and] different ways to cook vegetables.
Behavior change	
	I think I am more conscious when it comes to actually reading labels and choosing what I need.
	I actually think I found myself cooking more.
	I cannot wait to make a veggie tray. That is part of my weekly staple, if not once, twice, three, four times a week, or I learned how to utilize leftovers in a unique way, whether I make soup or something else, or add it to a salad, or add a few greens to it, which I never ever would have done before.
Challenges	
	I would have to figure out how to position [the] tablet that I was using, so that you could see what I was doing when I was cooking [which] was kind of difficult.
	It was hard because you didn’t really get to know your peers … the other participants in class.
	The hard thing was people would start talking and then you all had to be quiet and then wait for, you know, and it was just zoom, zoom is hard to do.
Logistics feedback	
	The only thing … that was hard for me was just making sure that I was picking up the food.
	It was just so peaceful being by myself in my own kitchen, setting everything up, not worrying about anything else and then waiting for the class to start.
	Overall impression, I mean you guys did a fantastic job. It was well organized, [and] the time management was fantastic.
Content feedback	
	Definitely the nutrition piece [was the most helpful], I can’t even speak more highly of all that and then bringing the mindfulness into it and tips and suggestions on how to prepare [by] watching the preparation of something.
	There were so many new things that I did learn, so many tricks, and putting it all together with the mindfulness, with the culinary skills and everything. It worked together perfectly.
	I think it was helpful to watch [the chef] demo how to put the meal together rather than just following exactly down the recipe. I thought that was just fun. [Chef’s] tips were great. And I think it was nice to hear other people’s inputs on their experiences or stories or how they did things.

## Data Availability

The original contributions presented in this study are included in the article. Further inquiries can be directed to the corresponding author.

## Abbreviations

BMI, body mass index; HbA1c, hemoglobin A1C; LDL, low-density lipoprotein; HDL, high-density lipoprotein; ALT, alanine aminotransferase.

## Appendix A. Curriculum Domains and Core Competencies

**Nutrition**
Evidence based nutrition recommendations
Healthy Plate, link between diet and health
Vegetables and fruits
Whole grains and carbohydrates
Healthy fats and oils
Proteins: beans and legumes, animal sources, seafood
Dairy and drinks
Fermented foods
Breakfast, lunch, dinner, dessert
Eating out and special occasions
Recipe adaptation
**Culinary Skills**
Knife skills, kitchen safety
Sautéing, stir-frying, searing
Grilling
Broiling
Roasting, baking
Steaming
Poaching
Boiling, simmering
Stewing, braising
Pressure/slow cooking
Emulsifying, whisking
Grating, zesting
**Culinary techniques**
Vegetable cookery
Whole grain and pasta cookery
Salads and vinaigrettes
Bean and legume cookery
Soups, stock, stews and curries
Yogurt-based sauces
Egg cookery
Meat cookery
Basic baking and whole food desserts
Adding flavor, herbs and spices
**Meal Planning and Food Shopping**
Meal planning and preparation
Navigating food retail locations (grocery stores, farmers’ markets, CSAs, etc.)
Reading food labels/using nutritional info
Strategies for saving money while food shopping
Limiting food waste through planning, shopping, storage
Stocking a pantry, food staples
Food storage and food safety
**Mindfulness, Mindful Eating, Stress**
Benefits of using mindful practices to foster self-awareness
Recognizing how mindful practices focus attention on action (such as eating and cooking)
Using mindful practices to reduce stress and improve well-being
Types of mindful practices (meditation, awareness, breathing, mindful eating)
Strategies for incorporating short mindful practices into one’s daily routine
Self-compassion
Happiness and joy
**Behavior Change Strategies**
Creating a wellness vision
Goal setting
Working with emotions
Problem solving
Planning for success, sustaining change
Small changes add up, persistence
**Physical Activity**
Balance and stretching
Strength
Aerobic activity
Link to health
**Sleep**
Basic sleep recommendations and link to health

## Appendix B. Curriculum Outline

**Week**	**Class Topic/Theme** **(Nutrition-Based)**	**Recipes (Serve 4)** **V = Vegetarian Option**	**Culinary Techniques**	**Culinary Skills** **Knife Skills in All**	**Goal Setting, Mindfulness & Exercise**
**1**	Building a healthy plate Nutrition overview Obesity as a disease Pillars of lifestyle change	Roasted veggies pasta Roasted veggies (separate recipe)	Pasta cookery Roasted veggies Flavor profiles, cuisines & cultures	Knife skills intro Boiling pasta Roasting veggies	Goal setting Introduction to SMART goals Wellness vision/why
**2**	Vegetables & fruits Sustainability, planetary impact	Farmer salad with seared Salmon V = Pan-roasted chick peas	Egg cookery Making vinaigrette Fish cookery Making salads	Emulsifying/whisking Blanching Searing/sautéing fish Boiling eggs	Mindfulness: introduction Mindful bite
**3**	Whole grains & carbs Glycemic index, carbohydrate quality	Farro salad Quick farro sauté	Grain cookery Making vinaigrette Making salads	Chopping nuts Toasting nuts Sautéing veggies Emulsifying/whisking	Exercise: introduction Flexibility fitness break
**4**	Healthy fats & oils	Tofu & veggie stir fry	Making a stir-fry	Stir-frying	Mindfulness: mindful meditation
**5**	Protein—beans & legumes fiber	Turkey veggie chili V = canned beans	Making chili/stew Yogurt sauces	Toasting spices Sautéing veggies	Exercise: aerobic fitness break
**6**	Protein—animal Planet health	Roasted chicken with root veggies—sheet pan dinner V = canned chick peas	Sheet pan meals	Roasting meat/veggies	Mindfulness: 3 min breathing
**7**	Protein—seafood	Fish taco with rainbow slaw V = canned black beans	Making tacos Making slaw Fish cookery Yogurt sauces Making vinaigrette	Searing/sautéing fish Emulsifying/whisking	Exercise: strengthening fitness break ideas
**8**	Dairy, drinks & fermented foods Sugars, fructose and artificial sweeteners	Infused waters and teas Yogurt parfait Grilled marinated tofu + asparagus Yogurt ranch dressing	Infusing water Cooking with tofu Yogurt sauces	Grilling	Midway goals
**9**	Shopping & label reading Spices & hearty greens	Chana masala Garlicky greens Lentils	Making curry/stew Using spices	Sautéing veggies	Exercise: balance fitness break
**10**	Breakfast Sleep & meal timing	Frittata (egg cups) Overnight oats V = Tofu scramble	Egg cookery (baked) hot cereals	Baking eggs	Mindfulness: body awareness exercise
**11**	Lunch & snacks *Managing stress*	Kale salad Tuna nicoise sandwich V = Chopped veggie + egg sandwich	Vinaigrette Hearty greens Toasting nuts Grain cookery Egg cookery Making salads	Boiling Emulsifying/whisking Baking/toasting Grating/Microplane	Exercise: flexibility fitness break
**12**	Dinner & shopping/meal planning	Roasted cauliflower & sweet potato Black bean turkey burgers V = Black bean, carrot & sweet potato burger	Roasted veggies Blended burgers Yogurt sauces	Roasting veggies Searing/grilling meats Chopping herbs/garlic	Mindfulness: 3 min breathing exercise
**13**	Eating out & special occasions	Red lentil pasta with marinara	Pasta cookery Making a sauce	Boiling	Exercise: aerobic fitness break
**14**	Desserts & added sugar	Walter Willett concept—chocolate, nut & fruit plate Baked apple Moroccan carrot lentil soup	Baked/grilled fruit Making soups Legume cookery	Baking fruit	Mindfulness: strengthening fitness break
**15**	Recipe modification Nutrition review	Cauliflower quinoa mac & cheese	Making casseroles Grain cookery	Baking Roasting veggies	Exercise: walking meditation exercise
**16**	Putting it all together: Lifestyle patterns	Grain bowl with choice of grain + protein + vegetable + sauce cook off!	Egg cookery Grain cookery Yogurt sauces	Blanching Searing/grilling meat/tofu	Final goals Reflect back to why/wellness vision & SMART goals

## Appendix C. Additional Survey Assessments

**Table A1:** Mindful Eating Questionnaire [70]: Baseline and post-intervention (*n* = 12).

Scores	Baseline Mean (SD)	Post Class Mean (SD)	*p*-Value
Awareness Sub Score	2.12 (0.68)	2.62 (0.71)	**0.01**
Distraction Sub Score	2.75 (0.25)	2.74 (0.58)	0.94
Disinhibition Sub Score	2.43 (0.63)	2.97 (0.71)	**0.01**
Emotional Sub Score	2.29 (0.74)	2.80 (0.73)	**0.01**
External Sub Score	2.34 (0.44)	2.56 (0.61)	0.20
**Total Score**	**11.94 (1.89)**	**13.68 (2.64)**	**0.01**

Note: boldface indicates significance.

**Table A2:** Prime Diet Quality Score 30 (PDQS-30) [69]: Baseline and post-intervention (*n* = 12).

Survey Question	Baseline Mean (SD)	Post Class Mean (SD)	*p*-Value
Over the past month, how often did you eat/drink … [≤once/month, 2–3 time/month, 1–2 times/week, 3–4 times/week, 5–6 times/week, once a day, ≥2 times/days]			
Q1. dark green leafy vegetables? Include fresh and cooked vegetables, eaten separately or as part of a mixed dish. Examples of foods: Spinach, kale, Swiss chard, arugula, collard greens, mustard greens, romaine lettuce, Bok choy	2.92 (0.79)	3.83 (1.19)	0.06
Q2. cruciferous vegetables? Include fresh and cooked vegetables, eaten separately or as part of a mixed dish. Examples of foods: Cabbage (white, red, Chinese), broccoli, Brussel sprouts, cauliflower, turnip, radish, rutabaga, kohlrabi	2.92 (1.0)	3.83 (0.83)	**0.01**
Q3. deep orange vegetables? Include fresh, frozen, canned, or thermally processed vegetables, eaten separately or as part of a mixed dish. Examples of foods: Carrot, pumpkin, sweet potato, dark orange squash such as butternut.	2.75 (1.14)	3.58 (1.0)	0.06
Q4. potatoes (mashed or baked)? Include when eaten separately or as part of a mixed dish. Do NOT include sweet potatoes, French fries or chips.	3.0 (1.28)	2.58 (0.90)	0.10
Q5. other vegetables? Include fresh and cooked vegetables, eaten separately or as part of a mixed dish. Examples of foods: Eggplant, tomato, pepper (e.g., green or red bell pepper), cucumber, onion, zucchini and beetroot.	3.5 (1.09)	4.42 (1.31)	0.11
Q6. citrus fruits? Include only whole fruit, not juices. Examples of foods in this group: Orange, grapefruit, tangerine, or other citrus fruit	1.92 (1.0)	2.92 (1.68)	0.06
Q7. deep orange fruits? Include fresh, frozen, canned, or thermally processed fruits, eaten separately or as part of a mixed dish. Include only whole fruit, not juices. Examples of foods: Mango, papaya, cantaloupe, apricot	1.5 (0.80)	1.58 (0.67)	0.67
Q8. other fruits? Include only whole fruit, not juices. Include fresh, frozen, or canned fruits. Examples of foods in this group: Apple, berries (e.g., strawberry, raspberry, blueberry), peach, plum, prune, grapes, avocado, pear or other fruits	3.25 (1.06)	4.08 (1.56)	0.06
Q9. beans, peas, and soy products? Include when eaten separately or as part of a mixed dish. Examples of foods in this group: Beans (e.g., pinto, navy, black, kidney beans, edamame, soy milk, tofu, tempeh), peas (e.g., green peas, chickpeas, hummus. Exclude peanuts) and lentils (e.g., red lentil, brown lentil, yellow lentil, other)	2.58 (1.08)	3.42 (1.16)	0.06
Q10. nuts and seeds? Include when eaten separately or as part of a mixed dish. Examples of foods in this group: Nuts (e.g., peanut, walnut, almond, pecan, pistachio, mixed nuts), seeds (e.g., pumpkin, sesame, sunflower seeds, other seeds) or nut or seed butter (e.g., peanut or sesame butter/tahini, other)	3.42 (1.44)	3.5 (1.57)	0.87
Q11. poultry? Do NOT include luncheon meats, hot dogs, organ meat, chicken nuggets, luncheon meat, and pâté. Examples of foods in this group: Chicken, turkey, duck and other poultry	2.83 (0.83)	3.25 (1.22)	0.27
Q12. fish? Include fresh, frozen, or canned. Do NOT include fried fish.	1.58 (0.79)	2.5 (0.67)	**<0.01**
Q13. red meat? Include muscle and organ meat as a main dish/as a part of a mixed dish. Examples of foods in this group: Beef, veal, lamb, goat, pork, other red meat	2.5 (1.09)	2.33 (1.07)	0.17
Q14. processed meats? Do NOT include very small quantities used for seasoning. Examples of foods in this group: Sausage, salami, bologna, pepperoni, ham, bacon, cured meat, beef jerky, corned beef, hot dog, frankfurter, chicken nuggets, canned meat	2.5 (1.08)	1.75 (0.62)	**0.04**
Q15. eggs? Include hen, duck, goose, or other poultry eggs. Examples of foods: Scrambled eggs, fried eggs, boiled eggs, quiche or similar egg-based dish	2.58 (0.90)	2.83 (0.83)	0.28
Q16. drink low-fat milk? Include products with 2% milk fat or less. Include only products made of animal milk.	3.83 (2.37)	4.08 (2.11)	0.49
Q17 *. full or whole-fat milk? Do NOT include ice cream, yogurt, or kefir. Include only products made of animal milk.	1.42 (0.79)	1.83 (1.19)	0.14
Q18 *. cheese?	3.92 (1.0)	3.5 (1.09)	0.05
Q19 *. fermented foods? Examples of foods: yogurt, kefir, kombucha, sauerkraut, kimchi	1.83 (1.27)	2.08 (1.51)	0.63
Q20. whole grains? Include cereals, porridges, pastas, breads, and baked goods containing at least 50% whole grain. Examples of foods in this group: Wholegrain bread or pasta, cereals (e.g., granola, muesli, Fiber 1, Kashi), plain popcorn, porridge (e.g., oatmeal, whole wheat, whole corn flour) or other whole grains (e.g., quinoa, amaranth, millet, brown rice, other)	3.42 (1.51)	4.17 (1.19)	0.06
Q21. refined grains and baked products? Examples of foods in this group: White bread, bagel, doughnut, bun or roll, white rice, couscous, noodles or pasta, pizza or pie crust, baked goods (e.g., pancake, cracker, waffle, muffin, tortilla, pita, matzo, naan), cereals (e.g., corn flakes, puffs, Chex cereal), Choco Pops, polenta	5 (1.54)	3.5 (1.24)	**0.01**
Q22 *. salty snacks? Examples of foods in this group: pretzels, potato chips, cheesy popcorn	3.42 (1.24)	2.67 (1.23)	**0.03**
Q23. sugar-sweetened beverages? Do NOT include coffee or tea, milk or cereal-based sugary drinks, homemade juices and diet drinks with artificial sugar. Examples of foods in this group: Sodas/soft drinks (e.g., Coca-Cola, Pepsi, Fanta, Sprite. Exclude diet sodas), energy drinks (e.g., Red Bull, Monster, and sport drinks with added sugar. Exclude sugar free drinks) and commercial fruit juices and fruit drinks with added sugar	2.25 (1.86)	1.33 (0.65)	0.11
Q24 *. alcoholic beverages?	2.0 (0.95)	1.92 (1.24)	0.59
Q25. sweets and ice cream?	4.75 (1.60)	3.67 (1.50)	**0.02**
Q26. fried foods?	2.5 (1.09)	2.08 (0.90)	0.05
Q27. [use] liquid oils in preparing your meals or seasoning salad? Do NOT include semisolid oils (e.g., palm and coconut oil) or solid fats	3.83 (1.64)	5.08 (1.38)	**0.01**

Note: boldface indicates significance. * Indicates questions added, with permission and input by original developers.
